# Genome-wide analysis of gene expression during *Xenopus tropicalis *tadpole tail regeneration

**DOI:** 10.1186/1471-213X-11-70

**Published:** 2011-11-15

**Authors:** Nick R Love, Yaoyao Chen, Boyan Bonev, Michael J Gilchrist, Lynne Fairclough, Robert Lea, Timothy J Mohun, Roberto Paredes, Leo AH Zeef, Enrique Amaya

**Affiliations:** 1 Faculty of Life Sciences, University of Manchester, Oxford Road, Manchester, M13 9PT UK; 2 The Healing Foundation Centre, Faculty of Life Sciences, University of Manchester, Oxford, Road, Manchester, M13 9PT UK; 3 MRC National Institute for Medical Research, Mill Hill, London, NW7 1AA UK

## Abstract

**Background:**

The molecular mechanisms governing vertebrate appendage regeneration remain poorly understood. Uncovering these mechanisms may lead to novel therapies aimed at alleviating human disfigurement and visible loss of function following injury. Here, we explore tadpole tail regeneration in *Xenopus tropicalis*, a diploid frog with a sequenced genome.

**Results:**

We found that, like the traditionally used *Xenopus laevis*, the *Xenopus tropicalis *tadpole has the capacity to regenerate its tail following amputation, including its spinal cord, muscle, and major blood vessels. We examined gene expression using the *Xenopus tropicalis *Affymetrix genome array during three phases of regeneration, uncovering more than 1,000 genes that are significantly modulated during tail regeneration. Target validation, using RT-qPCR followed by gene ontology (GO) analysis, revealed a dynamic regulation of genes involved in the inflammatory response, intracellular metabolism, and energy regulation. Meta-analyses of the array data and validation by RT-qPCR and *in situ *hybridization uncovered a subset of genes upregulated during the early and intermediate phases of regeneration that are involved in the generation of NADP/H, suggesting that these pathways may be important for proper tail regeneration.

**Conclusions:**

The *Xenopus tropicalis *tadpole is a powerful model to elucidate the genetic mechanisms of vertebrate appendage regeneration. We have produced a novel and substantial microarray data set examining gene expression during vertebrate appendage regeneration.

## Background

Humans have a limited capacity to regenerate, and thus, severe injuries result in unsightly scarring, loss of function and disfigurement (reviewed in [1]). Some vertebrates, however, possess remarkable capacities to regenerate complex body parts following injury (reviewed [2,3]). For example, certain newts and salamanders completely regenerate limbs, tails, jaw, and eye lens following removal (reviewed in [4]). Frogs, particularly during their larval tadpoles stages, have remarkable capacities to regenerate tissues following traumatic injury (reviewed in [5,6]). Despite ongoing investigation, we still lack a clear molecular understanding of the mechanisms and pathways responsible for vertebrate appendage regeneration.

In recent years, the *Xenopus *tadpole tail regeneration model has emerged as a powerful system for the study of vertebrate appendage regeneration (reviewed in [7,8]). The *Xenopus *tadpole tail represents a particularly interesting regenerating appendage, as it contains many axial and paraxial tissues, including the spinal cord, notochord, dorsal aorta, and skeletal muscle, of which all regenerate following amputation. Elegant studies using this model have uncovered important roles for FGF, Wnt, BMP and TGFβ signaling during tail regeneration [9-12]. In addition, this system has been valuable in elucidating additional mechanisms involved during tissue regeneration, such as the role of extracellular components, apoptosis, transcription factors and electrical signals [13-17]. Given the complexity of regeneration, however, it is likely that many important genes and cellular processes during *Xenopus *tail regeneration remain unknown.

The primary aim of our study, therefore, was to measure gene expression changes during regeneration of the *Xenopus tropicalis *tadpole tail in a genome-wide fashion. In particular, we sought to create a gene expression data set to serve as a resource in identifying the genes and processes involved in tail regeneration of this species. Although a similar study has been done previously in *X. laevis*, we chose to pursue this study in *X. tropicalis*, since this system contains more advanced genomic resources [18]. For example, unlike *X. laevis*, *X. tropicalis *is diploid and possesses a sequenced genome, making most genetic analyses simpler [19,20]. Notably, the sequenced genome of *Xenopus tropicalis *facilitated the creation of a genome-wide Affymetrix microarray chip based on more than 1.2 million ESTs and gene models from the *X. tropicalis *genome [19,21]. Despite these extensive genomic resources, tail regeneration in *X. tropicalis *had not been previously documented [6].

Here, we characterized the regenerative response that follows tadpole tail amputation in *Xenopus tropicalis*. We then catalogued the changes in the mRNA transcriptome during three different phases of regeneration using the Affymetrix *Xenopus tropicalis *genome array in biological duplicate, and thus, created a novel mRNA transcriptomic resource examining vertebrate wound healing and regeneration. Ultimately, the *Xenopus tropicalis *tadpole tail regeneration model, transcriptomic dataset, and the subsequently identified genes and processes implicated during regeneration will help facilitate current and future studies of vertebrate appendage regeneration.

## Results

### The *Xenopus tropicalis *tadpole has the capacity to regenerate its tail

**Figure 1:**
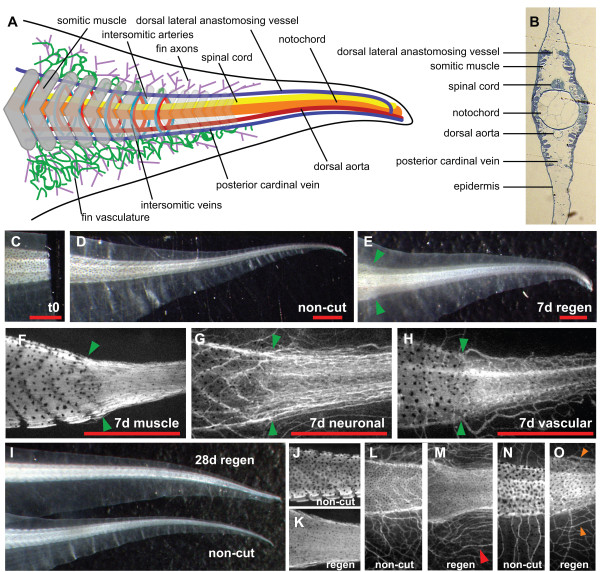
**The *Xenopus tropicalis *tadpole has the capacity to regenerate its tail**. (**A**) Schematic diagram of the tissues located in the *Xenopus tropicalis *tadpole tail. (**B**) Transverse section of tadpole tail visualized with toluidine blue. (**C-E**) An amputated tail (C), uncut tail (D), and regenerated tail 7 days after amputation (E). (**F-H**) Immunostaining against skeletal muscle (12/101; F), neurons (acetylated tubulin; G), and vasculature (mTie-2::eGFP transgene; H). (**I-O**) A regenerated tail at one month post-amputation (I). Immunostaining showing skeletal muscle (12/101; J, K), neurons (acetylated tubulin; L, M), and vasculature (mTieGFP transgene; N, O) in non-cut and regenerated tails 28 days post amputation. Green arrowhead depicts amputation site; red arrowhead shows parallel axonal tract; orange arrowheads shows parallel blood vessels. Red scale bar is 1000 μm.

Since tail regeneration in *Xenopus tropicalis *had not been previously described, we initiated this study by characterizing the regenerative capacity of this model following tail amputation (schematic diagram and transverse section of tadpole tail tissues are shown in Figure 1A-B). We amputated the tails of pre-metamorphic tadpoles (stages 49-51, [22]) and found that within one week, 95% of tadpoles regenerated tail appendages (N = 20, Figure 1C-E,). At seven days post amputation, the interface between the original tail and the regenerated portion of the tail was still discernible under bright field microscopy, due to a difference in optical density between the original versus regenerated portions of the tail (16 of 19 tails, green arrow, Figure 1E). Hence, we sought to address whether this apparent difference was due to a qualitative difference between the original tissues versus the regenerated tissues in the tails. We therefore stained the tails with acetylated tubulin and 12/101 monoclonal antibodies, which recognize differentiated neurons and skeletal muscle, respectively. To detect the vasculature, we created a transgenic *Xenopus tropicalis *line that transcribes eGFP under the control of the murine Tie-2 promoter. We found that this line expresses eGFP in the three major tail blood vessels (the dorsal aorta, posterior cardinal vein, and dorsal lateral anastomosing vessel), fin vasculature, as well as a small subset of circulating blood cells (Additional file 1, Figure S1 and Additional file 2, Movie S1).

Using these resources, we found that the regenerated tails differ in certain respects, compared to non-amputated tails. The most obvious difference in the regenerated portion of the tail was the loss of segmentation of somitic muscle and intersomitic axons (10 of 10 cases, Figure 1F-G), a phenomenon also reported in *X. laevis *[5]. The interface between the regenerated and unwounded vasculature also showed an increased amount of blood vessels in the regenerated portion (Figure 1H). The regenerated vasculature was found to be functional as defined by a return of circulation to the dorsal aorta, posterior cardinal vein, and dorsal anastomosing vessel (data not shown).

At one month post amputation, we observed that the regenerated tail was able to grow in size similar to a non-amputated control tail (Figure 1I). However, the normal, chevron patterning of the muscle did not return to the amputated portion of the tail (Figure 1J vs 1K, N = 6). Furthermore, at one month post amputation, we found that the perpendicular extending "branched" pattern of neurons in a non-amputated tail was replaced by neurons running parallel to the tail body in the regenerated tail (9 of 10 cases, Figure 1L-M, red arrow head). This replacement of "branched" by parallel patterning was also seen in fin vasculature of the tail (7 of 10 cases, Figure 1N-O, orange arrowhead). Despite this, however, the tails regained total functionality, and the tadpoles were able to swim indistinguishably to non-amputated sibling tadpoles. Taken together, these data show that the *Xenopus tropicalis *tadpole has the capacity to regenerate its tail appendage following amputation and restore full functionality, although tissue patterning of the regenerated tail is not identical to the non-amputated tail.

### Characterization of the early, intermediate, and late phases of tail regeneration

**Figure 2:**
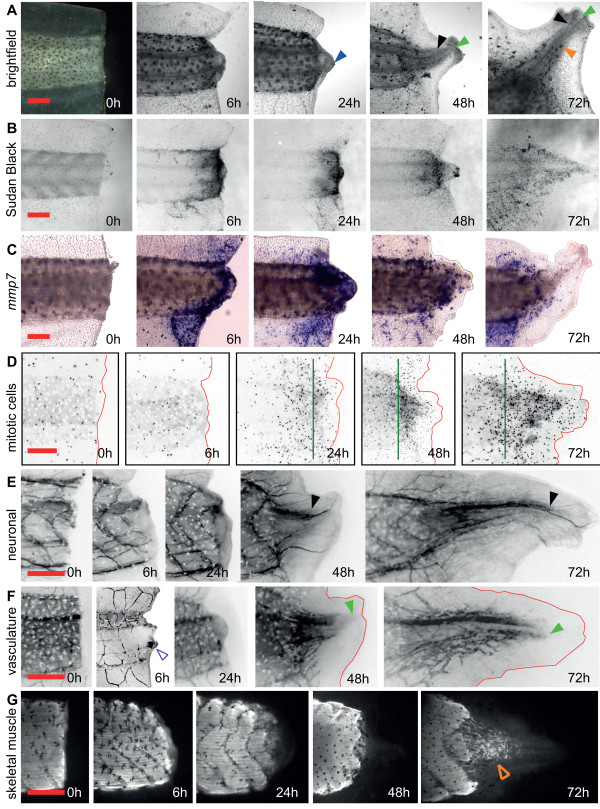
**Characterization of the early, intermediate, and late phases of tail regeneration**. (**A**) Tadpole tails imaged using bright field microscopy at early (0 h, 6 h); intermediate (24 h) and late phases (48 h and 72 h) of tail regeneration. Black open arrowhead shows typical dorsal constriction at 6 h post amputation; blue arrowhead shows pre-regenerative tissue; red arrowhead shows nascent fin epidermis at the distal tip; black arrowhead = spinal cord; green arrowhead = notochord, orange arrowhead = melanophrore. (**B**) Tadpole tails stained with Sudan Black B (inflammatory cells). (**C**) Tadpole tails stained by whole-mount *in situ *hybridization for *mmp7 *(inflammatory cells). (**D**) Tadpole tails stained by immunohistochemistry for mitotic cells (pH3, shown in black). The tail in each panel is outlined in red and the plane of amputation is shown in green. (**E**) Tadpole tails stained to reveal the neuronal tissue by immunohistochemistry (acetylated tubulin, shown in black). (**F**) Tadpole tails stained by immunohistochemistry for vascular tissue (GFP antibody in mTie-2::eGFP transgenic line, shown in black). The open purple arrowhead shows a typical eGFP positive "clot" at the injured dorsal aorta. Green arrowheads show distally projecting blood vessels. (**G**) Tadpole tails stained by immunohistochemistry for muscle tissue (12/101). The open orange arrowhead shows the presence of skeletal muscle in the regenerated portion of the tail. Red scale bar for each row is 250 μm. Note that the images in panels D-F are black and white reversals (i.e. shown as negative images).

We next sought to characterize the early (6 hours post amputation, or hpa), intermediate (24 hpa) and late (48-72 hpa) phases of tail regeneration [10]. Assessment of gross morphology (Figure 2A, N = 20 for each time point) showed that by 6 hpa, bleeding had ceased in all tails, and that by 24 hpa, 65% of tails possessed a 50 μm or greater amount of nascent tissue distal to the notochord (Figure 2A, blue arrow). By 48 hpa, all tails showed a regenerating notochord and spinal cord (Figure 2A, green and black arrow respectively). At 72 hpa, the average notochord length had doubled, and 85% of tails possessed melanophores in the regenerated portion of the tail (Figure 2A, orange arrow head).

We next assessed the presence of an inflammatory-like response following amputation, a response known to occur during tail and limb regeneration [23-25]. Our approach was to use the neutrophil granule histological stain, Sudan Black [26,27], or whole-mount *in situ *hybridization for *mmp7*, a marker gene for myeloid cells [27-29]. These data showed a marked increase in Sudan Black and *mmp7 *staining from 0 to 6 hpa that was largely concentrated in muscle-bearing portion of the tail (12/12, Figure 2B,C) Sudan Black and *mmp7 *staining cells remained at 24 hpa (16/17, Figure 2B,C) but diminished by 48 and 72 hpa (12/16 and 14/17 respectively, Figure 2B,C). These data suggest that an inflammatory-like response is activated by 6 hpa and remains for several days.

We then addressed cell proliferation by staining regenerating tails with a monoclonal antibody against phospho-histone H3, a marker of cells undergoing mitosis [30]. Immunohistochemistry using this antibody showed relatively few dividing cells at 0 and 6 hpa at the site of amputation (N = 7, Figure 2D). However, by 24 hpa, a localized increase in dividing cells was detected at the site of amputation (6 of 8 tails, Figure 2D), a pattern that continued at 48 and 72 hpa (8 of 8, and 7 of 7 tails, Figure 2D). These data demonstrated a localized increase in cell proliferation at the site of amputation during the intermediate (24 hpa) and late (48-72 hpa) phases of tail regeneration.

We next sought to assess the timing of overt regeneration of the nascent neural, vascular, and muscle tissues. Using the acetylated tubulin monoclonal antibody, we detected spinal cord regeneration (closed black arrowhead) and axons (open red arrowhead) at 48 hours post amputation (Figure 2E). These neurons projected axons into the distal most portion of the regenerating tail. To address the vasculature, we imaged the mTie-2::eGFP transgenic line and found that by 6 hpa, 17 of 20 tails examined had an eGFP positive "clot" at the injured portion of the dorsal aorta, possibly originating from the eGFP positive cells present in the blood circulation in this line (Figure 2F, open purple arrowhead). Blood vessels extended into the regenerating tail tip between 24 and 48 hpa (Figure 2F, 20 of 20 cases, closed green arrowheads). Lastly, new skeletal muscle cells began to differentiate between 48 and 72 hpa (Figure 2G, open orange arrowhead).

In summary, characterization of the early, intermediate, and late stages of *X. tropicalis *tail regeneration pointed to distinct biological activities in these time periods: an inflammatory-like phase by 6 hpa (early phase), a cell proliferation phase beginning 24 hpa (intermediate phase), and a regrowth phase of differentiated tissues, such as neurons, notochord, muscle and vasculature by 48 and 72 hpa (late phase).

### Transcriptomic analysis of the early, intermediate, and late stages of tail regeneration

**Figure 3:**
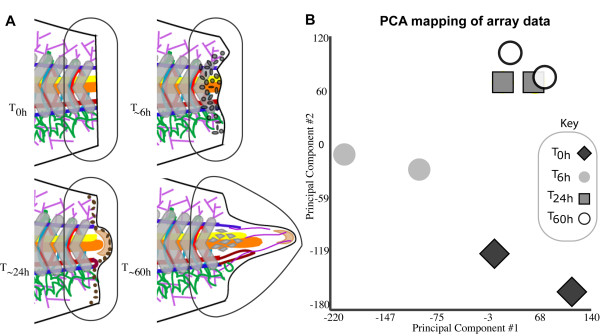
**Array analysis of *X. tropicalis *tail regeneration**. (**A**) The schematic diagrams of the early, intermediate, and late stages of tail regeneration. The circled areas depict the portion of tissue, and hence the RNA, collected and used for array analysis. (**B**) Shows the similarities of the eight arrays (four array time points in duplicate) using principal component analysis (PCA) mapping.

We next endeavored to catalogue the changes in the transcriptome through the early, intermediate, and late phases of regeneration using the Affymetrix *Xenopus tropicalis *genome array. Our approach was to collect RNA from the site of amputation/regeneration during the early, intermediate, and late stages of tail regeneration as well as an equivalent T_0h _reference sample (Figure 3A). We repeated this experiment twice, using different pools of tadpole tail tissues. The resulting four array groups are referred to by their mean post amputation collection time: T_0h _(reference), T_6h _(early regeneration), T_24h _(intermediate regeneration), and T_60h _(late regeneration). Microarray analysis was carried out using the MAS5.0 algorithm and two-sample t-tests, resulting in both a p-value and the more stringent q-value for each probe set (q-value is a statistical measure similar to p-value, but one that takes into consideration false discovery rate) [31].

As a first step in our analysis, we sought to assess the similarities between the arrays in a global fashion. To establish relationships and compare variability between replicate arrays, principal component analysis (PCA) was used. PCA was chosen for its ability to reduce the effective dimensionality of complex gene-expression space without significant loss of information [32]. We performed PCA mapping with our eight arrays (four array time points in duplicate) and found that, importantly, equivalent array duplicates clustered with one another (Figure 3B). This analysis also showed that the most significant gene expression changes occurred between the T_0h _vs T_6h _and the T_6h _vs T_24h _time periods (Figure 3B). In contrast, the T_60h_ vs T_24h _array were almost indistinguishable using these analytical conditions. The largest number of significant gene expression changes and the largest fold change magnitudes occurred in the T_6h _vs. T_0h _comparison; there were 422 targets that possessed an over 5-fold change (up or down) accompanied by a q-value of under .05 in the T_6h _vs T_0h _comparison, while only 20 targets fit these stringency conditions in the T_60h _vs. T_24h _comparison (Table 1).

**Table 1:** Analysis *X.tropicalis *array

		Array Comparison	
	**6 h vs 0 h**	**24 h vs 6 h**	**60 h vs 24 h**
**A. Number of targets with given q-value**			
q < .01	124	12	0
q < .05	2236	936	55
**B. Number of q-value < 0.05 targets with given expression level change**			
> 5× up	314	58	8
> 2× up	833	237	19
> 2× down	675	290	20
> 5× down	108	89	12
**C. Average expression level change amongst the top 20 targets up or down regulated with q-value < 0.05**			
fold up	40.5	17.1	5.2
stdev	9.3	9	3.9
fold down	17.1	18.8	8.2
stdev	9.0	7.6	8.8

In order to analyze the array experiment, we next annotated the array using a modified BLAST approach and successfully enriched the annotation nearly twofold (Additional file 3, Figure S2). Analyzing this new annotation, we found a considerable amount of gene target redundancy in the array. Of the 58,861 targets in the array, we found that there were 16059 unique RefSeq protein IDs (Supplemtary Figure 2B). From these, we next created a data set that included the most significant target for each RefSeq protein ID and analyzed the expression activity of these targets throughout the four measured time points (Additional file 4, Figure S3). These data showed that ~45% of genes targets showed at least one expression level change of greater of less than 2 fold between successive time points. We further analyzed and validated some of the most dynamically expressed genes in a later section of this manuscript.

Using these annotations, we compared our *X. tropicalis *array results to data previously reported in a cDNA macro-array analysis of *X. laevis *tail regeneration [23]. To link the NIBB *X. laevis *clones used in the macro-array analysis carried out by Tazaki et al. (2005), we first performed protein-protein tblastx searches of the NIBB clones against the most recent NCBI protein databases. In this way, we linked 47 of the 77 NIBB clones reported in the previously published cDNA macroarray [23] to targets in our Affymetrix array using common gene homologues. The Tazaki et al. (2005) data examined gene expression at 36 hours and 72 hours post amputation versus a 0 hour post amputation control. We compared these expression level change values to their closest equivalent in our array experiment, namely, the expression level changes stemming from our T_24h _vs T_0h _and T_60h _vs T_0h _comparisons. These analyses showed that, out of the 47 *X. laevis *comparisons, 39 (T_24h _vs T_0h_) and 36 (T_60h _vs T_0h_) *X. tropicalis *comparisons were in agreement in terms of up or down regulation (Additional file 5, Figure S4). Moreover, the highest fold upregulated *fgf *gene in our array data was *fgf20*, which we also validated with by RT-qPCR and whole-mount *in situ *hybridization (data not shown), consistent with findings in *X. laevis *[11]. These data strongly suggest that the molecular mechanisms responsible for tail regeneration are conserved between the allotetraploid *X. laevis *and the diploid *X. tropicalis*.

Taken together our data represent a characterization of the transcriptomal changes which occur during the early, intermediate, and late phases of tail regeneration, thereby creating the most comprehensive catalogue of gene expression data examining *Xenopus *tail regeneration to date. The entire array dataset can be found in the MIAME compliant standard to the Array Express database (Experiment E-MEXP-2420).

### Validation and analysis of highly significant array targets

**Figure 4:**
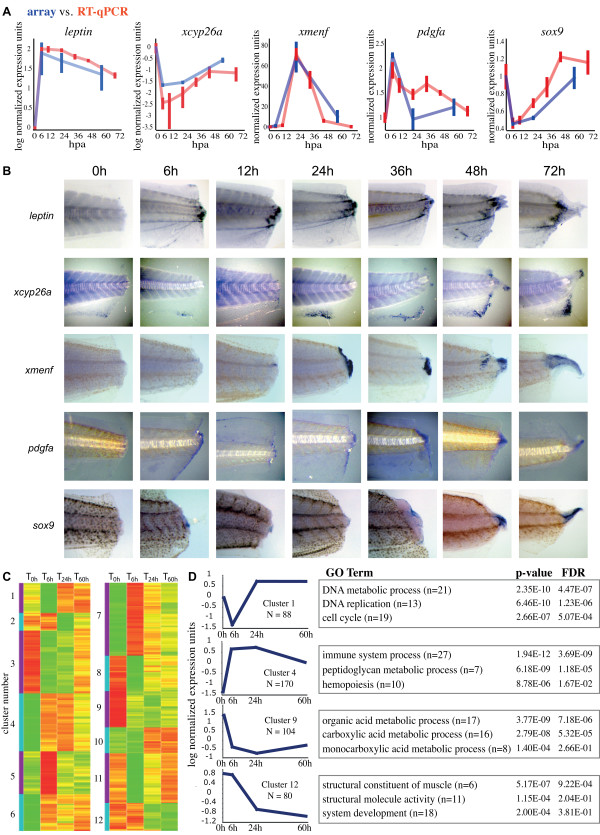
**Validation and GO analysis of highly significant targets**. (**A**) The validation of five highly significant array targets (blue) is plotted against normalized RT-qPCR derived expression profiles (red). Note that lines connecting each time point are meant to serve as a visual aide. (**B**) Shows expression patterns of RT-qPCR validated targets using whole-mounts *in situ *hybridization for *leptin*, *xmenf*, *xcyp26a*, *sox9 *and *pdgfa *at 0 h, 6 h, 12 h, 24 h, 36 h, 48 h, and 72 hours post-amputation. (**C**) Heat map of all highly significant and dynamic targets produced a group of 1441 targets, grouped into 12 clusters by their similar expression changes through the array time points. (**D**) Gene ontology (GO) analysis of the twelve clusters in (C) showed four clusters over-represented with at least three GO terms accompanied by a p-value under 1e-3 and false discovery rate (FDR) under 0.5. Error bars represent standard error of mean (SEM).

We next chose to validate by RT-qPCR five targets possessing a q-value < 0.05 and at least a two-fold expression level change. Three of the targets chosen for validation possessed the highest significant magnitude changes in their respective groups (*leptin*, *xcyp26a*, *xmenf*); the other two possessed a more modest fold change of ~2-fold (*pdgfa, sox9*). For RT-qPCR, new RNA samples were collected in biological triplicate at 0 h, 6 h, 12 h, 24 h, 36 h, 48 h, and 72 hours post amputation. Resulting qPCR gene Ct values were compared to Ct values of reference gene *rps18*. The comparison of gene expression values to another reference gene *rpl8 *yielded similar results (data not shown). By normalizing array and RT-qPCR expression values with regards to their T_0h _values (e.g. T_0h _expression value = 1 relative expression unit), we found that 5 of 5 of these targets with q-value < 0.05 possessed a strong quantitative correlation between array and RT-qPCR derived expression values (Figure 4A, blue versus red, note that lines connecting time points are meant to serve as a visual aide). We also assessed the expression of these genes by whole-mount *in situ *hybridization: these data showed upregulations of *leptin*, *xmenf*, *pdgfa, and xcyp26a *in agreement with the array and RT-qPCR results (Figure 4B).

We next sought to analyze all highly significant targets contained in the array dataset using clustering and gene ontology (GO) [33]. Target validation using RT-qPCR (Figure 4A) led us to examine all targets in our array dataset with high significance (q-value under 0.05) and dynamic expression level changes (at least one expression level change greater than 2 fold up or down). Applying this stringency to the array data produced a list of 1441 probe sets (corresponding to 1024 unique genes), which were then grouped into 12 clusters with regards to their similar expression changes through the array time course (Figure 4C). To determine whether these clusters represented functionally similar genes, we examined BP (biological processes) and MF (molecular function) GO term representation between each gene cluster and the entire array using the DAVID bioinformatic database [34]. Four of the twelve clusters were over-represented with at least three GO terms accompanied by a p-value under 1e-3 and a false discovery rate under 0.5 (Figure 4D). Cluster 4, a group of targets that showed upregulation in the T_6h _array, was over represented with genes involved in the immune/defense response; these data were in agreement with our analysis of the early phase of regeneration using Sudan Black staining and *mmp7 in situ *hybridizations (Figure 2B-C). Similarly, cluster 1 were over represented with genes implicated in the cell cycle and DNA replication during the intermediate phase of regeneration; these data were in agreement with our results on cell proliferation (Figure 2D). Also, cluster 12, which showed downregulation during the intermediate and late phases of regeneration, was over represented with genes associated with muscle differentiation; this result is probably due to the fact that the tissue collected for these array samples possessed proportionally less differentiated muscle tissue than the T_0h _and T_6h _arrays (Figure 2G). Finally, cluster 9, which showed a downregulation during the early, intermediate, and late regeneration phases, was over represented with genes associated with organic acid metabolism.

### Systematic analysis of metabolic processes

Our initial, unbiased analysis of the most statistically significant array targets led us to a cluster that were over represented with genes involved in organic acid metabolism (Figure 4D). Given that only 17 of the 104 targets in that cluster actually possessed the "organic acid metabolism" GO term, we wondered if all genes involved in "organic acid metabolism" would show a similar pattern of gene regulation. Hence, we next took a biased approach and asked if other intracellular metabolic processes would show concerted patterns of regulation during tail regeneration. For this reason, we systematically examined the expression profiles of all intracellular metabolic processes present in our array data set.

**Figure 5:**
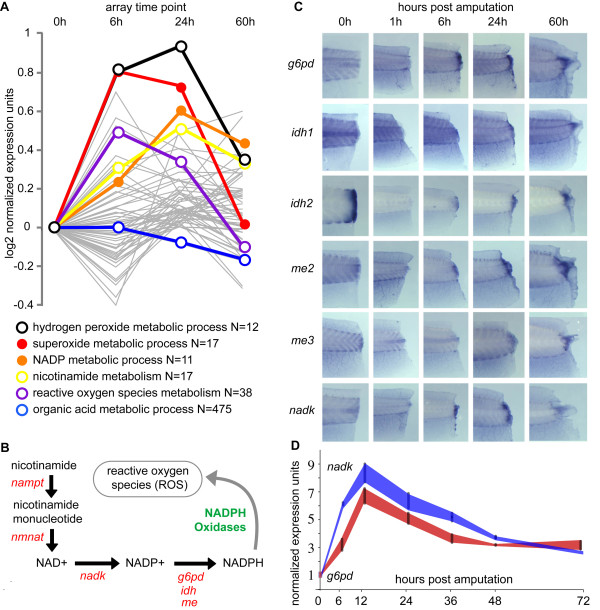
**Systematic analysis of intracellular metabolic processes using gene ontology**. (**A**) 66 metabolic processes are upregulated in the T_24h _array (gray). Five of ten highest upregulated processes in the T_24h _array are colored. The key to this graphic is shown below and "N" indicates the number of genes in the array corresponding to each process. "Organic acid metabolism", which was identified in an initial GO analysis (Figure 4D) is also plotted on the graph. (**B**) Nicotinamide, NADP, and the generation of reactive oxygen species (ROS) are connected by the metabolic pathway shown in (B) (genes regulating the pathway shown in red, proteins in green). (**C**) Shows the *in situ *hybridization patterns of *g6pd, idh1, idh2, me2, me3*, and *nadk *following tail regeneration (0 h, 1 h, 6 h, 24 h, and 60 h). (**D**) RT-qPCR expression profile of *nadk *(blue) and *g6pd *(red) following tail amputation. Error bars represent standard error of mean (SEM), lines connecting each error bar are meant to serve as a visual aide.

Our approach for pan-array intracellular metabolic processes analysis was to link each gene to its respective metabolic processes as defined by the Gene Ontology Consortium [33] and screen these metabolic processes for their average log2 expression profiles through T_6h_, T_24h _and T_60h _arrays. In total, this approach led to the examination of 155 intracellular metabolic processes (Additional file 6, Figure S5). In this method of analysis, unlike the analysis shown in Figure 4D, the 475 gene targets that entailed "organic acid metabolic process" did not show a marked change in expression level during regeneration (Figure 5A).

We then decided to focus our attention on the intracellular metabolic processes that were upregulated during the early to intermediate phases of tail regeneration, a time when the immune response is evident and cells begin to proliferate, but precede the overt regeneration of differentiated tissues (Figure 2). In total, 66 processes were upregulated in the T_24h _vs T_0h _comparison (Figure 5A) and we were interested to find that "hydrogen peroxide metabolic process", "superoxide metabolic process", "NADP metabolic process", "nicotinamide metabolic process", "oxygen and reactive oxygen species metabolic process" were amongst the top ten most upregulated processes in the T_24h _vs T_0h _comparison (Figure 5A).

Intriguingly, nicotinamide, NADP, and the generation of reactive oxygen species (ROS) and hydrogen peroxide are connected via the generation and subsequent oxidation of reduced nicotinamide adenosine dinucleotide phosphate, also known as NADPH (Figure 5B). To further assess whether genes governing the NADPH metabolic pathway were activated following tail amputation, we assessed via whole-mount *in situ *hybridization the expression of the NADP/H synthetic genes shown in Figure 5B, of which *g6pd*, *nadk*, *me2*, *me3*, *idh1*, and *idh2 *showed expression changes following tail amputation and during regeneration using this technique (Figure 5C). We confirmed the expression level changes via RT-qPCR of *nadk *and *g6pd*, and these data showed an upregulation of ~9-fold (*nadk*) and ~6-fold (*g6pd*) by 12 hours post amputation (Figure 5D). The modulation of these NADP/H related genes suggest a role for NADPH dependent metabolic processes during tail regeneration.

## Discussion

At the initiation of this study, the regenerative capacity of the *Xenopus tropicalis *tadpole tail had not yet been reported, though one study had shown that, like *X. laevis*, *X. tropicalis *can regenerate its lens following removal [35]. The primary aims of this report, therefore, were to characterize tail regeneration in *X. tropicalis *and create a transcriptomic resource that would allow the identification of genes and processes that are modulated during vertebrate tissue repair and regeneration. Here, we have shown that in a manner that appears very similar to *X. laevis*, the *X. tropicalis *tadpole tail can also regenerate its tail, including notochord, spinal cord, skeletal muscles and the major blood vessels (dorsal aorta and posterior cardinal vein).

In order to examine the regeneration of the vasculature, however, we established a new transgenic line, the mTie-2::eGFP transgenic line. Analysis of regenerated vascular and neuronal tissue demonstrated that both the fin axons and blood vessels adopted a "parallel" morphology. Furthermore, we found that neurons extend axons in this "parallel" manner to the distal most portion of the regenerating tail by 48 hpa. These data suggest a possibility that the neurons, which extend further into the regenerating tail, provide guidance to the regenerating blood vessels, as has been suggested during development [36,37]. The distal, pioneering activity of the nerves during regeneration is consistent with their critical role during limb and tail regeneration [3,38-40].

While *X. tropicalis *and *X. laevis *frog species are thought to have diverged at least 30 million years ago [41,42], our data suggest a strong similarity in gene expression during the tadpole tail regeneration between these two species. However, one advantage of *X. tropicalis *as a model is its sequenced genome, which facilitated the generation of a comprehensive Affymetrix genome array. Using this resource, we examined the mRNA transcriptome of the early, intermediate, and late phases of *X. tropicalis *tail regeneration, identifying over 1000 highly dynamic genes.

Despite the large number of genes found in this analysis, it cannot be considered a completely comprehensive study, as it is based on duplicate arrays from pooled samples obtained at four different time points. A more comprehensive microarray analysis would have required analysis of many more samples from individual animals during various stages of tail regeneration. This, however, was not possible as we are able to isolate RNA only once from each tadpole. In light of this, we instead pooled samples of tail tissue for each array, which allowed an average analysis of many individual animals in duplicate. A deeper, more comprehensive analysis of the transcriptome would require a higher number of arrays, and possibly, a means to extract mRNA at multiple time points of regeneration from single individuals.

An example of how this genome-wide array helped identify novel genes involved in tail regeneration was *leptin*, a gene, which lacked EST evidence in *Xenopus tropicalis*. Nevertheless, the *leptin *gene possessed the highest significant upregulation in the T_6h _vs. T_0h _array comparison in our array dataset. *Leptin*, first discovered in mice and dubbed "the obesity gene", due to the phenotype presented in mutant mice [43], has subsequently been shown to act as a chemokine, which regulates metabolism. Leptin has also been shown to act as an angiogenic factor during wound healing and is potent activator of JAK/STAT signaling, acting downstream of the Leptin receptor ([44], reviewed in [45]). Moreover, *Xenopus *and mammalian Leptin appear to function in physiologically similar ways e.g. by influencing feeding behavior as well as stimulating cell proliferation in the developing limb [46]. It remains to be determined to what extent Leptin acts as an angiogenic factor, a growth factor, or a modulator of energy consumption during tail regeneration.

Another intriguing result from the array experiment was the identification of *cyp26a*, a gene encoding a p450 cytochrome that metabolizes retinoic acid. Retinoids are important signaling molecules and morphogens implicated in a wide variety of developmental and regenerative processes, including *X. laevis *limb regeneration [47-49]. While *cyp26a *possessed the greatest significant decrease in fold change in the T_6h _vs T_0h _array comparison, it also showed a striking increase in expression in the T_60h _vs T_24h _array comparison. This dynamic regulation, which we validated via RT-qPCR, suggests that a tightly controlled regulation of retinoic acid levels is important during regeneration. In the embryo, *cyp26a *has been previously shown to be important in the establishment of boundaries and give positional information to developing tissues, as well as interacting and modulating the Fgf pathway [50] (reviewed in [51]).

Yet another interesting gene identified in our screen was *Xmenf*. Transcripts for this gene showed rapid upregulation following wound healing, peaked during early regeneration, and then decreased during tail regeneration. The exact function of *xmenf *is currently unknown, though in *Xenopus *embryo, *xmenf *expression is upregulated prior to gastrulation, and in combination with *Xenopus *nodal related 2 (Xnr2), can potentiate the induction of ventral mesoderm in the animal cap cells [52]. Intriguingly, the *xmenf*, and closely related *xenf*, do not appear to possess mammalian orthologues [53].

GO analysis of highly significant gene targets identified the modulation of genes involved in aspects of metabolism following tail amputation. Reanalysis of array data with a focus on intracellular metabolic processes identified the modulation of a set of genes that regulate the generation of NADP/H. This focused, meta-analysis demonstrated the utility of our *X. tropicalis *microarray dataset as a resource for the identification of genes and biological processes that are modulated during vertebrate appendage regeneration. The oxidized and reduced forms of NADP are known to be important in a wide variety of biological processes [54]. For example, the oxidation of NADPH by the NADPH oxidase complex (NOX) is a critical step in the generation of reactive oxygen species (ROS), which can regulate several processes, including cell proliferation, differentiation [55,56], and the inflammatory response [57]. Conversely, NADPH is also essential in the maintenance of the endogenous antioxidant defense mechanisms e.g. the regeneration of reduced glutathione (GSH) from oxidized glutathione (GSSH) [58]. Moreover, the NADP+/NADPH ratio, along with other redox couples, helps define the redox state of the cell, which has long ranging consequences on gene transcription, protein activity, and phosphorylation.

## Conclusion

This report is the first to show that the *X. tropicalis *tadpole, like *X. laevis*, is a powerful vertebrate model for the study of appendage regeneration. The sequence genome of *X. tropicalis *allowed genome-wide transcriptomic characterization of multiple phases of tail regeneration thus producing a novel and extensive resource for identifying genes and processes important during appendage regeneration. It is hoped that discoveries made in the *Xenopus *model will facilitate the development of novel therapies that will promote healing and regeneration in humans.

## Methods

### *Xenopus tropicalis *tail amputation

For tail amputation, stage 49-51 tadpoles [22] were anesthetized with 0.1% MS222 (Sigma) in 0.01× MMR, and tails were amputated perpendicular to the notochord using a surgical blade (Swann-Morton #10). After amputation, tadpoles were allowed to recover to swimming behavior in a tank of fresh aquarium water and then placed into a clean, oxygenated aquarium (25°C). All animal procedures complied with the UK Animal (Scientific Procedures) Act 1986 and were conducted with UK Home Office approval, ref. PPL 40/3181.

### Microscopy

Confocal microscopy was performed with an Olympus Fluoview FV1000 imaging system and accompanying software, with the exception of Additional file 1; Figure S1A, which was taken using a Zeiss LSM 700 confocal microscope system. Imaging of bright field, whole-mount *in situ *hybridizations, and whole mount immunofluorescence were performed using a Leica MZ APO dissecting stereomicroscope or a Leica MZ FLIII fluorescent stereomicroscope and Northern Eclipse software (Empix, Canada). Images of stained sections were performed on an Olympus IX70 inverted microscope with the Northern Eclipse software (Empix, Canada).

### Generation of transgenic mTie-2::eGFP

Transgenic *X. tropicalis *embryos were generated as described [59] with modifications [60]. The *mTie2-*GFP construct [61] was linearized with *Sal I*.

### Histo- and immunohisto-chemistry

Toluedine blue staining was performed as described in [62], sections were cut at 1 μm thickness. Sudan Black B staining was performed in accordance with the company's protocol (Sigma-Aldrich). For immunohistochemistry, samples were fixed in MEMFA and antibodies were diluted 1:1000 in BBT (1 × PBS, 1% BSA, 0.1% Triton X-100) with 5% heat-treated lamb serum. The primary antibodies used were; anti-PH3 (Upstate), 12/101 [63], anti-acetylated tubulin (Sigma), anti-GFP (Sigma). Secondary antibody was used at 1:2000 (goat anti-mouse Alexa488 Invitrogen). For whole-mount *in situ *hybridization, DNA plasmids used to create DIG probes were collected from the *Xenopus tropicalis *EST library [21] or IMAGE consortium: *sox9 *(TNeu111f21), *xcyp26a *(TEgg054j08), *xmenf *(TNeu076n12), *pdgfa *(TEgg039p14), *nadk *(IMAGE:7003620), *g6pd *(TEgg077i08), *idh1 *(IMAGE:5307685.), *idh2 *(TNeu118i04), *me2 *(TNeu077n19), *me3 *(TGas021g06)*, mmp7 *(IMAGE 7005633). The DNA plasmid for *leptin *was produced by RT-PCR with primers ATGCAATATATTCACCTCTCAGTC (forward) and TTAGCAGTCAGTGATGTGG (reverse) and cloned into pTOPO CR2.1 (Invitrogen). DIG probes were generated from linearized DNA plasmids using 10xDIG labeling nucleotide mix and T7 RNA polymerase (Roche). Probes were purified using MicroSpin-50 columns (BioRad). Whole-mount *in situ *hybridization was performed according to Ho and Whitman (2008) [10].

### Affymetrix array analysis

Tissues samples (~250 μm proximal to the site of amputation) were excised and placed in ice-cold RNAlater. RNA was extracted using the RNA Easy Mini kit, with tissue homogenization performed with syringe and Qiashredder (Qiagen). The T_0h _array was composed of RNA from 60 tail pieces taken at the time of tail amputation, the T_6h _array was composed of RNA from 60 tail pieces taken at 6 h post amputation, the T_24h _array was composed of RNA from tail pieces taken at 12 h, 24 h, and 36 h post amputation (20 per time point), the T_60h _array was composed of RNA from tail pieces taken at 48 h and 72 h post amputation (30 per time point). These samples were collected from two batches of tadpoles, generated from separate matings and processed in duplicate. Technical quality control was performed with dChip (V2005) (http://biosun1.harvard.edu/complab/dchip/); [64]) using the default settings. Expression analysis and detection calls were performed with the MAS5.0 algorithm in Bioconductor [65], Affymetrix Microarray Suite User Guide, version 5 edition, 2001). Principal component analysis (PCA), batch removal, two-sample t-tests and false discovery rate correction with the q-value method [31] were performed on logarithm base 2 scaled data with Partek Genomics Solution (version 6.4). Fold changes were calculated using the mean average between array probesets. Microarray data has been submitted in a MIAME compliant standard to the Array Express database (Experiment E-MEXP-2420, http://www.ebi.ac.uk/arrayexpress).

### Array gene annotation

To maximise the numbers of Affymetrix *Xenopus tropicalis *genome array target probes associated with RefSeq protein IDs, we searched the probe set consensus sequences directly against NCBI protein data sets, and indirectly against the same protein sequences via a set of assembled *Xenopus tropicalis *EST gene sequences [21]. We downloaded the probe set consensus sequences for the array from Affymetrix (http://www.affymetrix.com/Auth/analysis/downloads/data/X_tropicalis.consensus.zip) and used BLASTx with an E-value cutoff of 10-3 to search these sequences against RefSeq protein sequences for human, mouse and fruit fly, and all GenBank protein sequences for *Xenopus tropicalis *and *Xenopus laevis*. The gene annotation file is available upon request.

### Expression profiling, target clustering, GO analysis, intracellular metabolic processes analysis

Expression profiling was performed by analyzing an array data set that included the most significant target for each RefSeq protein ID based on q-value (lowest sum of log(q-value T_6h _vs T_0h_)+ log(q-value T_24h _vs T_6h_)+ log(q-value T_60h _vs T_24h_)). Clustering was performed on a gene list of filtered probe sets from the T_6h _vs T_0h _or T_24h _vs T_6h _or T_60h _vs T_24h _comparisons (q-value < 0.05, fold change > ± 2.0 and present detection call in at least 1 of the 8 arrays) using K-means clustering with Manhattan Distance metric ("Super Grouper" plugin of maxdView software, http://www.bioinf.manchester.ac.uk/microarray/maxd/index.html). GO analysis was performed with DAVID (http://david.abcc.ncifcrf.gov/) using our improved human RefSeq protein ID annotation [34]. Metabolic processes were analyzed by taking the dataset that included the most significant target for each RefSeq protein ID and normalizing their expression values such that [T_0h] _= 1 relative expression unit. A list biological process and molecular function GO processes (and their respective RefSeq protein IDs) and were downloaded from DAVID, and intracellular metabolic processes possessing 10 or more genes in the array data set were examined.

### RT-qPCR

Tail tissues were collected in biological triplicate at 0 h, 6 h, 12 h, 24 h, 36 h, 48 h, and 72 h post amputation and placed into RNAlater (Qiagen). Tissue was homogenized with syringe and QIAshredder (Qiagen), total RNA was extracted using Qiagen's RNA Easy Mini kit, and cDNA was synthesized using Applied Biosystem's High Capacity cDNA kit. Taqman assays were generated to target exon-exon boundaries and qPCR reactions were run with Taqman Fast Gene Expression Master Mix on a StepOne+ qPCR machine (Applied Biosystems). Expression values were calculated using the ΔΔCt method with genes *rps18 *used as reference, error was calculated using standard error of the mean (SEM). Primers and probes are shown in Table 2.

**Table 2:** RT-qPCR Primer and Probe Sequences

PCR Primer Pair/Probe	Sequence
*X. tropicalis* *g6pd*	F: 5-GCAACATCAAGGAGACCTGCAT-3 R: 5-CGAAAGGTTTCTCCACGATAACAC-3 FAM: 5-TTCCAGCCCACAGAGCT-3
*X. tropicalis* *leptin*	F: 5-CAAAGATGTTGGCAAGGACCTT-3 R: 5-GCTCATCTGGGATAAAATCCAAACCA-3 FAM: 5-ACCCGATCCAGTTCCTT-3
*X. tropicalis* *mmp7*	F: 5-TGGAAATGCAGATATATTCATCCGATTCG-3 R: 5-ACCATAGGCGTGAGCTAAAACTC-3 FAM: 5-CATGCGTGCGTGCTC-3
*X. tropicalis* *nadk*	F: 5-GCACCAGGGAGCACCAA-3 R: 5-GGTTACTGGGCATGGTCCAT-3 FAM: 5-CTTGTACGCCTGAATTC-3
*X. tropicalis* *pdgfa*	F: 5-GCAGCGTCTCCTGGACATT-3 R: 5-GTTGGCTCCAGAAGCATCCT-3 FAM: 5-CCTCCTCCTACGGAATC-3
*X. tropicalis* *rps18*	F: 5-CAGACAGAAGGACATTAAGGATGGAA-3 R: 5-TCGCTCTAAATCTTCACGGAGTTT-3 FAM: 5-CAGCCAGGTTCTTGCC-3
*X. tropicalis* *sox9*	F: 5-GCATGAGCGAAGTCCACTCT-3 R: 5-AGGCTGGACGTCTGTCTTG-3 FAM: 5-ACTGACCTGAATGTTCTCC-3
*X. tropicalis* *xcyp26a*	F: 5-TGCCCTTCTTTGGAGAGACTCT-3 R: 5-CGTACTTCCTTCGCTTAACTTGGA-3 FAM: 5-TTGCGCCTCTGCAGCAC-3
*X. tropicalis* *xmenf*	F: 5-GCTGAAGCAGTGCAGAAACAG-3 R: 5-GCTTACAAAGTCCTCCTCATCTGA-3 FAM: 5-ACAGGGCCTACCCATCG-3

## List of abbreviations

ESTs: expressed sequence tags; PCA: principal component analysis; GO: gene ontology; hpa: hours post amputation.

## Authors' contributions

NRL performed most experiments, prepared data and the figures, and co-wrote the manuscript. YC assisted with immunohistochemistry, whole-mount *in situ *hybridization, imaging, nucleic acid purification and RT-qPCR. BB assisted with immunohistochemistry and RL carried out sectioning. RP assisted with imaging. TJM and LF generated the mTie-2::eGFP *X. tropicalis *line. MG performed the array gene annotations. LAHZ performed the array bioinformatic analysis and clustering. EA guided the project and co-wrote the manuscript. All authors read and approved the final manuscript.

## Supplementary Material

Additional file 1 **Figure S1 - A transgenic *Xenopus tropicalis *line that expresses eGFP in its vasculature**. **(A) **A series of merged confocal images from transgenic *Xenopus tropicalis *tadpole expressing eGFP under the control of the murine Tie-2 promoter; the four red boxes show the heart **(B**), brain and associated vasculature **(C)**, the vasculature that surrounds the eye **(D**), and the vasculature in the tail **(E)**. The single confocal slice in (E) depicts the dorsal lateral anastomosing vessel (l), spinal cord (s), dorsal aorta (d), posterior cardinal vein (p), epithelial vasculature (e), and intersomitic vessels (i). Click here for file

Additional file 2 **Movie S1 - Movie showing circulating, eGFP+ cells in the mTie-2::eGFP *X. tropicalis *transgenic line**. Click here for file

Additional file 3 **Figure S2 - An improved array gene annotation**. (**A-B**) The graphics show the increase in annotation rate from the company provided annotation (A) and our improved annotation (B). The number of annotated probe sets is represented by the area of the squares. Click here for file

Additional file 4 **Figure S3 - Sequential gene expression changes of all gene targets in array dataset**. The graphic maps the expression profiles of all 16059 RefSeq genes in the array data set. The area of the circles represents the number of genes in each respective expression level change group. Each subsequent node represents the transition of a set of genes from one array time point to the next (T_0h _- T_6h _- T_24h _- T_60h_). Between nodes, red lines represent a positive fold change of over two-fold between array time points, while blue lines represent a negative fold change over two-fold between array time points, and black lines represent a fold change that is between positive 2 and negative 2. In the end, this graphic allows one to track the expression level changes of all gene targets in the array. For example, from the T_0h _to T_6h _array, 2351 of the 16059 targets had an over two-fold increase in expression (indicated by the red line). Of these 2351 targets, 77 then had another over two-fold increase in the T_6h _to T_24h _array (indicated by the red line). Of these 77 targets, only 2 targets had an over two-fold increase in the T_24h _to T_60h _array (indicated by the red line). Click here for file

Additional file 5 **Figure S4 - Comparison of *X. tropicalis *microarray data to *X. laevis *macroarray**. The graphic plots the expression level changes reported in a previous *X. laevis *cDNA macro array (y-axis) versus the *X. tropicalis *data of this report (x-axis). The graphic was made by plotting the log2 expression level changes of the 47 targets from the *X. laevis *cDNA macro array that were also measured in our *X. tropicalis *array data and plotted. There are two comparisons shown on the graph, a comparison between the expression level changes comparisons of *X. laevis *D3/D0 post-amputation and *X. tropicalis *T_60h_/T_0h _(blue circles) and the expression level comparisons of *X. laevis *D1.5/D0 post-amputation and *X. tropicalis *T_24h_/T_0h _data (red squares). Click here for file

Additional file 6 **Figure S5 - Log2 expression profiles of intracellular metabolic processes**. The graphic shows the average log2 expression profiles of all 155 intracellular metabolic processes present in the array data. By ranking the processes by their T_6h _vs T_0h _expression level change, the 1st, 2nd, 3rd, 4th, and 5th quintiles of the 155 intracellular metabolic processes are colored red, orange, green, blue, and purple respectively. Click here for file
