# Supplementary figures and images for: Genome-wide analysis of gene expression during Xenopus tropicalis tadpole tail regeneration
Source: BMC Dev Biol. 2011 Nov 15;11:70. doi: 10.1186/1471-213X-11-70 (PMC3247858; doi:10.1186/1471-213X-11-70)

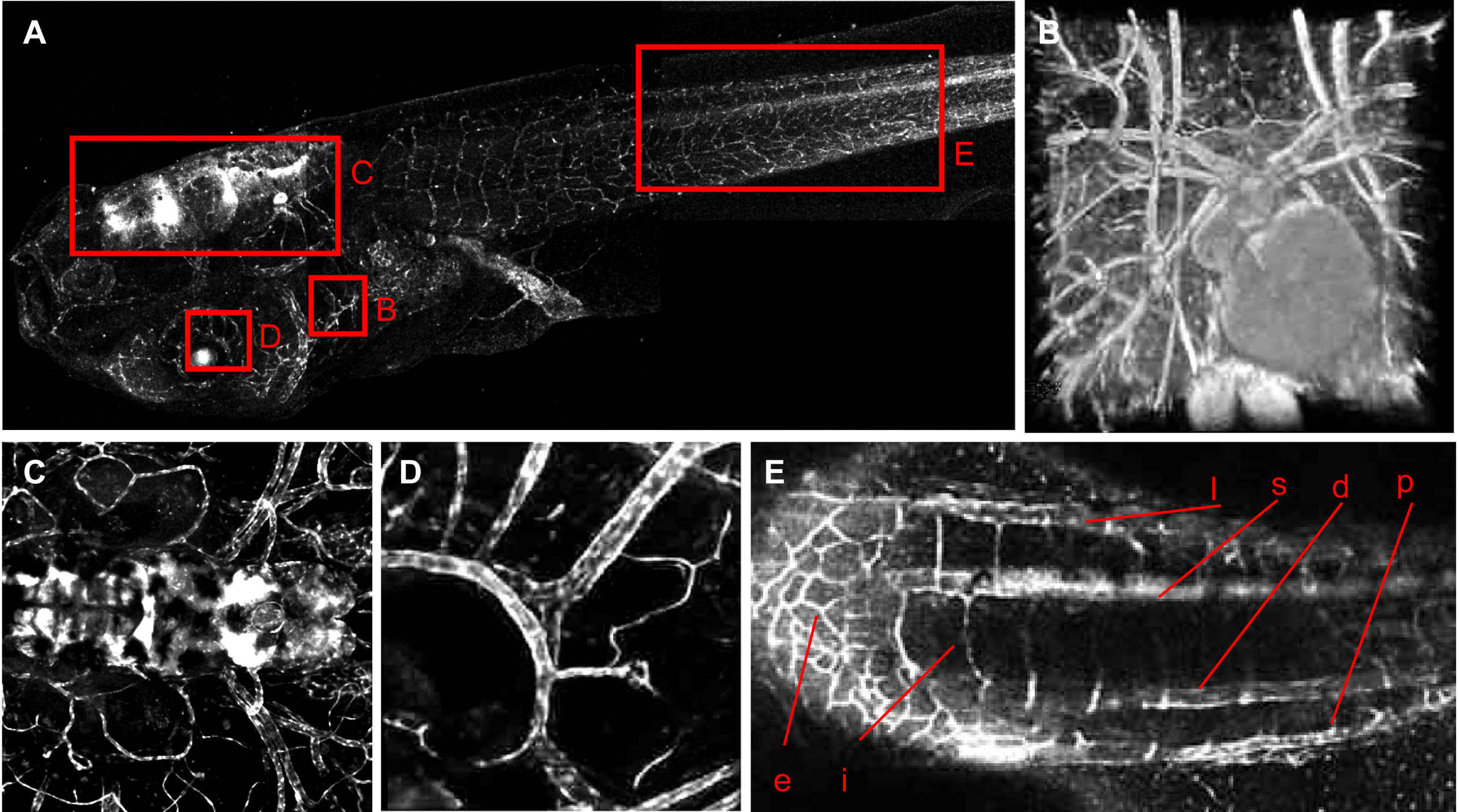

Supplement: Additional file 1 — Figure S1 - A transgenic Xenopus tropicalis line that expresses eGFP in its vasculature. (A) A series of merged confocal images from transgenic Xenopus tropicalis tadpole expressing eGFP under the control of the murine Tie-2 promoter; the four red boxes show the heart (B), brain and associated vasculature (C), the vasculature that surrounds the eye (D), and the vasculature in the tail (E). The single confocal slice in (E) depicts the dorsal lateral anastomosing vessel (l), spinal cord (s), dorsal aorta (d), posterior cardinal vein (p), epithelial vasculature (e), and intersomitic vessels (i). [file 1471-213X-11-70-S1.JPEG]

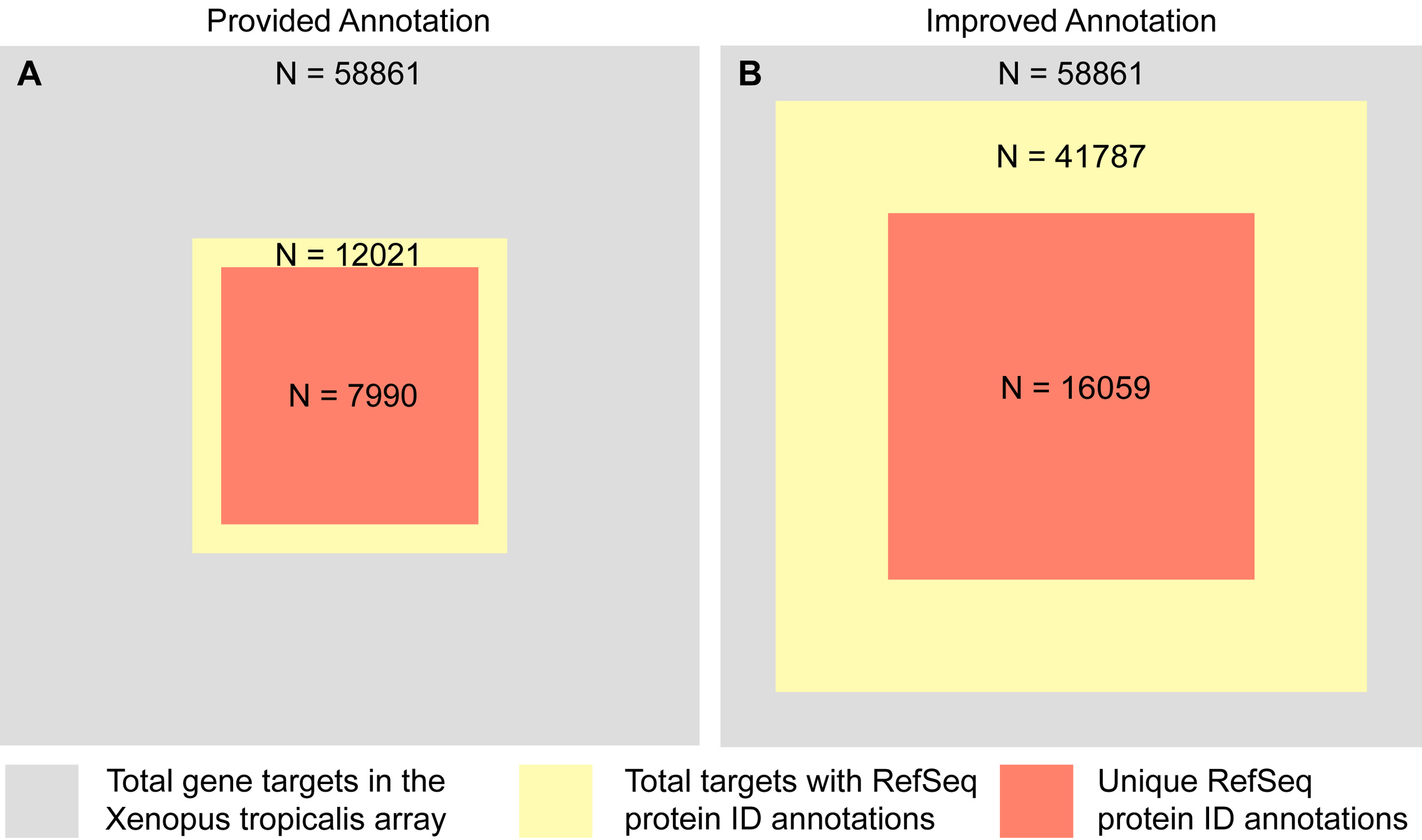

Supplement: Additional file 3 — Figure S2 - An improved array gene annotation. (A-B) The graphics show the increase in annotation rate from the company provided annotation (A) and our improved annotation (B). The number of annotated probe sets is represented by the area of the squares. [file 1471-213X-11-70-S3.JPEG]

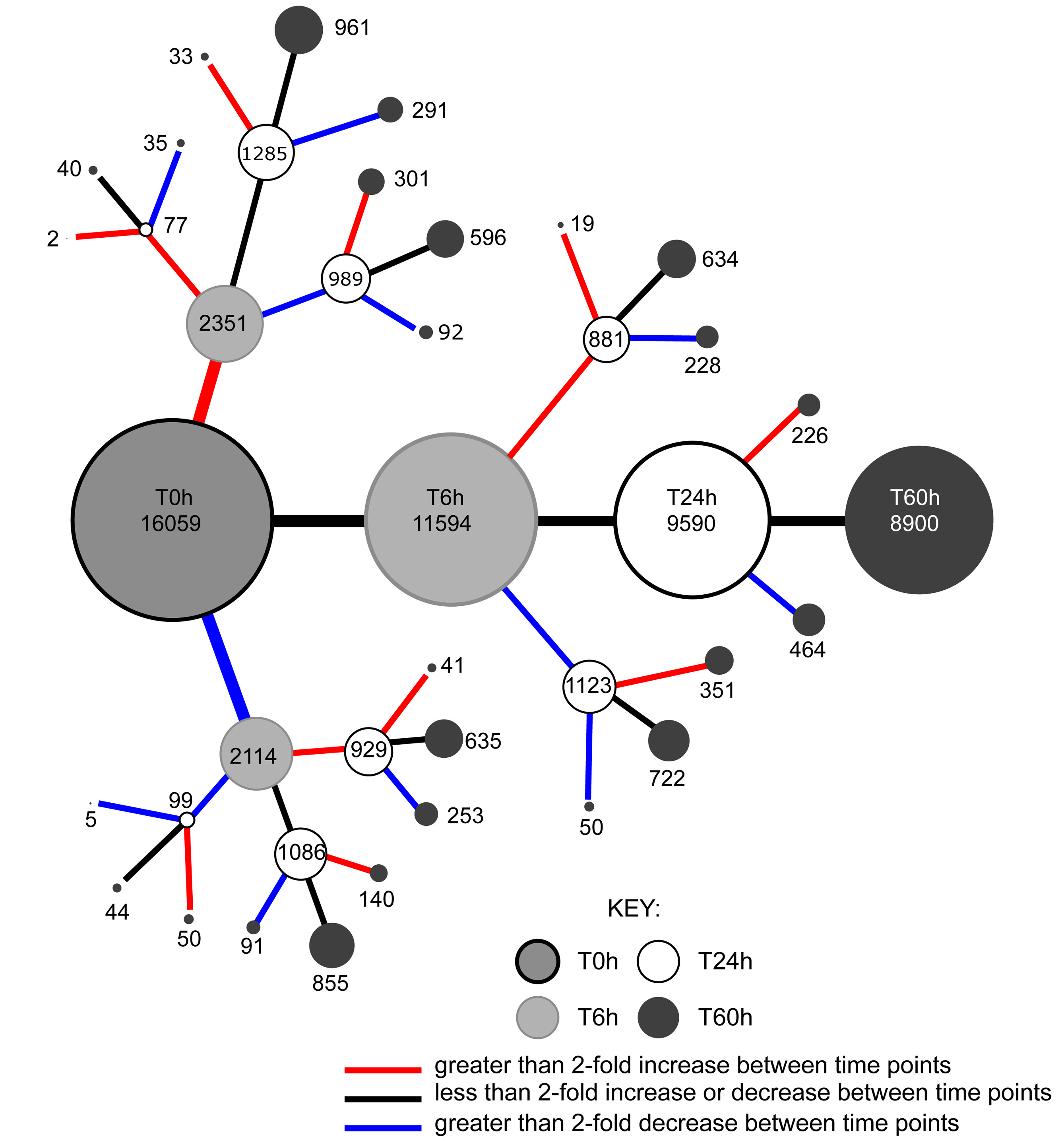

Supplement: Additional file 4 — Figure S3 - Sequential gene expression changes of all gene targets in array dataset. The graphic maps the expression profiles of all 16059 RefSeq genes in the array data set. The area of the circles represents the number of genes in each respective expression level change group. Each subsequent node represents the transition of a set of genes from one array time point to the next (T0h - T6h - T24h - T60h). Between nodes, red lines represent a positive fold change of over two-fold between array time points, while blue lines represent a negative fold change over two-fold between array time points, and black lines represent a fold change that is between positive 2 and negative 2. In the end, this graphic allows one to track the expression level changes of all gene targets in the array. For example, from the T0h to T6h array, 2351 of the 16059 targets had an over two-fold increase in expression (indicated by the red line). Of these 2351 targets, 77 then had another over two-fold increase in the T6h to T24h array (indicated by the red line). Of these 77 targets, only 2 targets had an over two-fold increase in the T24h to T60h array (indicated by the red line). [file 1471-213X-11-70-S4.JPEG]

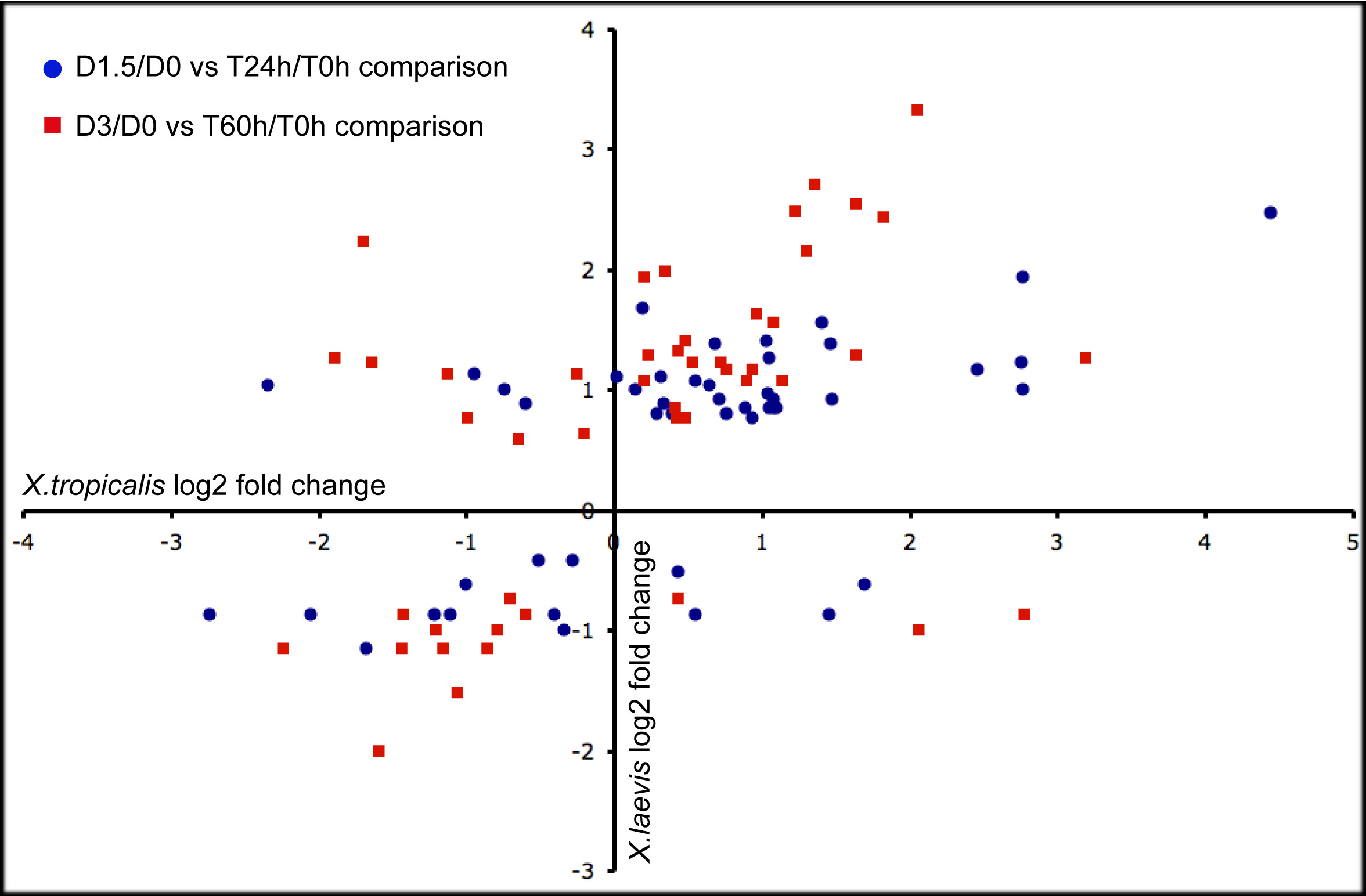

Supplement: Additional file 5 — Figure S4 - Comparison of X. tropicalis microarray data to X. laevis macroarray. The graphic plots the expression level changes reported in a previous X. laevis cDNA macro array (y-axis) versus the X. tropicalis data of this report (x-axis). The graphic was made by plotting the log2 expression level changes of the 47 targets from the X. laevis cDNA macro array that were also measured in our X. tropicalis array data and plotted. There are two comparisons shown on the graph, a comparison between the expression level changes comparisons of X. laevis D3/D0 post-amputation and X. tropicalis T60h/T0h (blue circles) and the expression level comparisons of X. laevis D1.5/D0 post-amputation and X. tropicalis T24h/T0h data (red squares). [file 1471-213X-11-70-S5.JPEG]

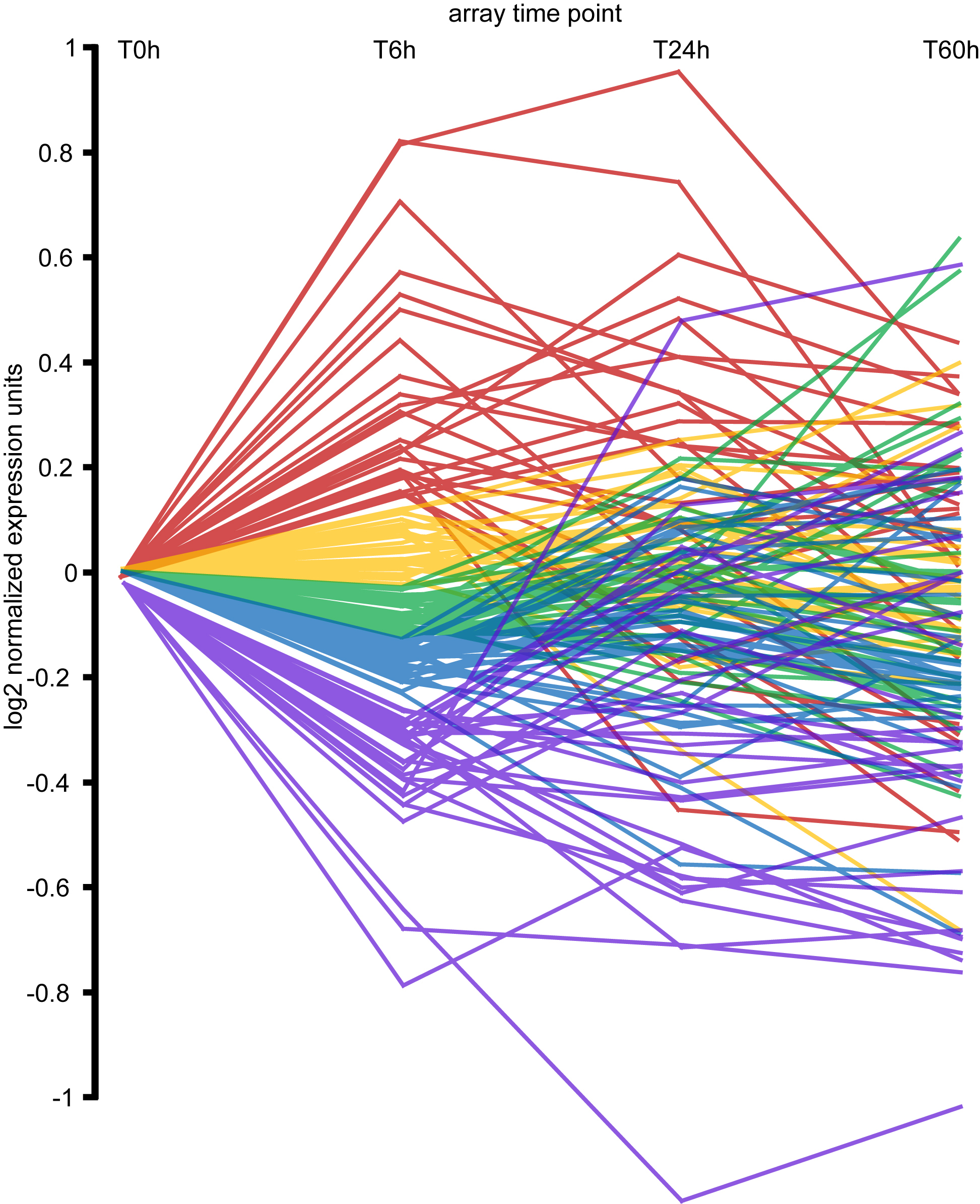

Supplement: Additional file 6 — Figure S5 - Log2 expression profiles of intracellular metabolic processes. The graphic shows the average log2 expression profiles of all 155 intracellular metabolic processes present in the array data. By ranking the processes by their T6h vs T0h expression level change, the 1st, 2nd, 3rd, 4th, and 5th quintiles of the 155 intracellular metabolic processes are colored red, orange, green, blue, and purple respectively. [file 1471-213X-11-70-S6.JPEG]
